# Fluid–structure interaction (FSI) simulation for studying the impact of atherosclerosis on hemodynamics, arterial tissue remodeling, and initiation risk of intracranial aneurysms

**DOI:** 10.1007/s10237-022-01597-y

**Published:** 2022-06-13

**Authors:** Ali A. Rostam-Alilou, Hamid R. Jarrah, Ali Zolfagharian, Mahdi Bodaghi

**Affiliations:** 1 Department of Engineering, School of Science and Technology, Nottingham Trent University, Nottingham, NG11 8NS UK; 2 School of Engineering, Deakin University, Geelong, 3216 Australia

**Keywords:** Atherosclerosis, Intracranial aneurysm, Hemodynamics, Aneurysm initiation, Biomechanics, Arterial tissue remodeling, Fluid–structure interaction (FSI)

## Abstract

The biomechanical and hemodynamic effects of atherosclerosis on the initiation of intracranial aneurysms (IA) are not yet clearly discovered. Also, studies for the observation of hemodynamic variation due to atherosclerotic stenosis and its impact on arterial remodeling and aneurysm genesis remain a controversial field of vascular engineering. The majority of studies performed are relevant to computational fluid dynamic (CFD) simulations. CFD studies are limited in consideration of blood and arterial tissue interactions. In this work, the interaction of the blood and vessel tissue because of atherosclerotic occlusions is studied by developing a fluid and structure interaction (FSI) analysis for the first time. The FSI presents a semi-realistic simulation environment to observe how the blood and vessels' structural interactions can increase the accuracy of the biomechanical study results. In the first step, many different intracranial vessels are modeled for an investigation of the biomechanical and hemodynamic effects of atherosclerosis in arterial tissue remodeling. Three physiological conditions of an intact artery, the artery with intracranial atherosclerosis (ICAS), and an atherosclerotic aneurysm (ACA) are employed in the models with required assumptions. Finally, the obtained outputs are studied with comparative and statistical analyses according to the intact model in a normal physiological condition. The results show that existing occlusions in the cross-sectional area of the arteries play a determinative role in changing the hemodynamic behavior of the arterial segments. The undesirable variations in blood velocity and pressure throughout the vessels increase the risk of arterial tissue remodeling and aneurysm formation.

## Introduction

Cardiovascular tissue remodeling that occurs near the brain and its neural tissues is the major cause of ischemic strokes. Among these, two chronic inflammatory reactions, atherosclerosis and aneurysm, are controversial stimuli that need more and more biological and physiological studies to be recovered clearly. In fact, ICAS, which accounts for 15% of all strokes, is a major concern for scientists (Gao et al. 2018). But IAs affect approximately 4%-6% of the general population (Sakarunchai et al. 2015). Even though more IAs remain clinically silent during their lifetime, the growth and development of some of them may appear as a ruptured lesion. Rupture of IAs leads to subarachnoid hemorrhage (SAH), which accounts for up to 7% of all strokes (Feigin et al. 2003). Subsequent SAH due to rupture in IAs influences 50% of mortality and 30%-50% of neurologic morbidity rates revealing the importance of studying novel diagnoses and therapy methods for IAs (Texakalidis et al. 2019). Also, Sato et al. (Sato et al. 2013) demonstrated that approximately one-fifth of patients with spontaneous intracranial hemorrhage had ICAS. However, the formation, growth, and rupture risk factors of IAs are determinative in the field, even though we have no complete and definite answer for pathogenesis and mechanism.

Although both phenomena have complex pathophysiology, the foremost problem is the fact that the relationship between atherosclerosis and aneurysm has not been completely revealed. But the influence of the common factors of smoking, hypertension, and familial predisposition has been demonstrated for both disorders (Nixon et al. 2010). Furthermore, the critical role of inflammation in the pathogenesis of these phenomena as a common link between them is undeniable (Feng et al. 2019). On the other hand, the hemodynamics of the aneurysm is affected by biomechanical and biological factors. One of these factors is atherosclerosis, which is caused by an increase in plaques over the years that leads to the narrowing of the vessel. This blockage reduces blood flow with local turbulence or sluggish perfusion. This is why understanding the relationship between aneurysms and atherosclerosis is important. Although endothelial dysfunction is the first step in the formation of an IA, some effective factors such as hemodynamic stress and vascular risk factors, along with some nonmodifiable factors including genetics, play a crucial role in this process (Krings et al. 2011; Etminan and Rinkel 2016).

Understanding the biomechanical effects and influence of mechanobiology features of ACAs have a close dependence on blood flow dynamics. In other words, hemodynamics plays a determinative role in studying the pathogenesis of atherosclerosis aneurysms. As a chronic inflammation, atherosclerosis appears due to the physiological response of the vascular wall to dyslipidemia and endothelial distress involving the inflammatory recruitment of leukocytes and the activation of local vascular cells. Then, the remodeling of the arterial vascular wall results in a resident's plaque development. The plaques may remain asymptomatic, a condition known as subclinical disease. Some of them become obstructive or stable angina, and a few are detected as thrombosis-prone or vulnerable (Riccioni and Sblendorio 2012). Mentioning plaque development (resulting in stenosis) disturbs the blood flow in the involving arteries and leads to a change in arterial wall mechanical stresses. The clump of plaques (fats, cholesterol, calcium, and other substances) in the inner layer of endothelium causes critical changes in hemodynamic responses of the corresponding artery.

It is worth to note that uncovering the involving biomechanical phenomena in the genesis of both aneurysm and atherosclerosis may open a door to finding early detection, therapy, and pharmaceutically conservative treatment methods. Besides, experimental and computational studies are carried out by researchers for different reasons such as hemodynamics and mechanical behavior of aneurysms, early prediction of inflammation initiation in arteries, and treatment methods and potentials (Jarrah et al. 2021). But some studies are performed to reveal strong evidence of pathophysiological and biomechanical relationships between IA and atherosclerosis. (Sugiyama et al. 2013) investigated hemodynamic characteristics of atherosclerotic lesions in IAs using CFD simulation. They reported the influence of the ACA wall on the rupture risk of IAs. Hokari et al. 2014 performed experimental and statistical studies to report that the impact of atherosclerotic factors on cerebral aneurysm formation depends on location. Leng et al. 2014 used CFD analysis to predict stroke recurrence in patients with an asymptomatic ICAS. In addition, the impact of atherosclerotic plaque mass and location of stenosis (Knight et al. 2010) on the hemodynamic responses of the atherosclerotic wall (He et al. 2017) is the other important biomechanical aspect of ACA. Also, the influence of hemodynamics on atherosclerotic changes in IAs was studied by Tateshima et al. 2008 for the first time. They showed how plaque deposition on the aneurysm wall can affect the dynamic behavior of the saccular section of an ACA in comparison with a non-atherosclerotic wall. Also, Fan et al. 2019 proposed a two-step method supported by both statistical analysis and CFD to study the morphometry and hemodynamics of coronary artery aneurysms caused by atherosclerosis. By presenting a comprehensive computational study, Ke et al. 2021 developed a mathematical model to mimic the progression of the ACA. Their model contained both the multi-layer structure of the arterial wall and the pathophysiology of the ACA to predict the progression of this kind of aneurysm. But the model was not able to show how an atherosclerotic lesion could be the cause of the initiation of an aneurysm. Although the impacts of atherosclerosis on location-related hemodynamics (Ahmadpour-B et al. 2021), arterial tissue degradation (Wang et al. 2022) and remodeling (Thon et al. 2018), and their effects on IAs have been investigated using FSI, but to the best of authors’ knowledge, no study has been conducted to investigate the biomechanical aspects of the initiation and formation of ACAs using an FSI simulation and analysis. Several studies conducted using CFD have demonstrated the pathological effects of blood flow on the biomechanics of atherosclerosis (Teng et al. 2021). On the other hand, some researchers have applied the FSI advantages in their investigations related to the plaque rupture and progression in atherosclerotic arteries (Ebrahimi and Fallah 2022), hemodynamic parameters assessment of cerebral aneurysms (Sun et al. 2022), the blood–artery interaction in abdominal aortic aneurysms (Kang et al. 2022), and investigation of the influence of postural changes on hemodynamics in the internal carotid artery bifurcation aneurysm (Ballambat et al. 2022) recently. The previous studies performed by arterial tissue researchers are organized for remodeling of the arterial tissue by local atherosclerosis plaque and the influence of the stenosis on hemodynamics. The impact of stenoses on the hemodynamics of brain arteries involving atherosclerosis has not been investigated in both physical and blood dynamics aspects. In fact, the question of, how atherosclerotic stenosis can affect the hemodynamics of a cerebral artery leading to arterial tissue remodeling considering the blood and tissue interaction, has not been answered clearly. However, the current study is carried out because of the obvious lack of FSI studies on biomechanical demonstration of the relationship between atherosclerosis and an IA with the pathophysiology of an artery.

The goal of the present study is multifold. For the first time, we provide a numerical study performed by an FSI analysis to investigate the impact of atherosclerosis on hemodynamics, initiation, and growth of an IA. Firstly, the corresponding cerebral artery bifurcations are created in three different scenarios: (i) without atherosclerosis (a healthy cerebral artery bifurcation), (ii) with stenosis (a cerebral artery includes ICAS), and (iii) with an ACA model (an atherosclerotic cerebral artery that with a saccular aneurysm in the bifurcation point). Then, the fluid dynamic responses of all models are observed and compared with each other by statistical and meta-analysis to show how an artery blockage affects the hemodynamics in a bifurcation. Finally, biomechanical results of the models are reported according to the nature of the aneurysm and atherosclerosis.

This paper is organized and divided into five sections. The first section gives a brief overview of IA initiation factors and their relationship with atherosclerosis, considering the blood flow conditions. The second section discusses effective biomechanical parameters and hemodynamics factors in the initiation and formation of an IA. In the third section, brief details of the FSI simulation and analysis methods are presented. We collect observations that show how an ACA may initiate in the fourth section. Finally, in the fifth section, the highlights and conclusions are presented. Due to the absence of similar results in the specialized literature, it is expected that the results of this research would contribute to a better understanding of the influence of atherosclerosis on hemodynamics, arterial tissue remodeling, and initiation risk of IAs.

## Effective biomechanical parameters and hemodynamics factors

**Fig. 1:**
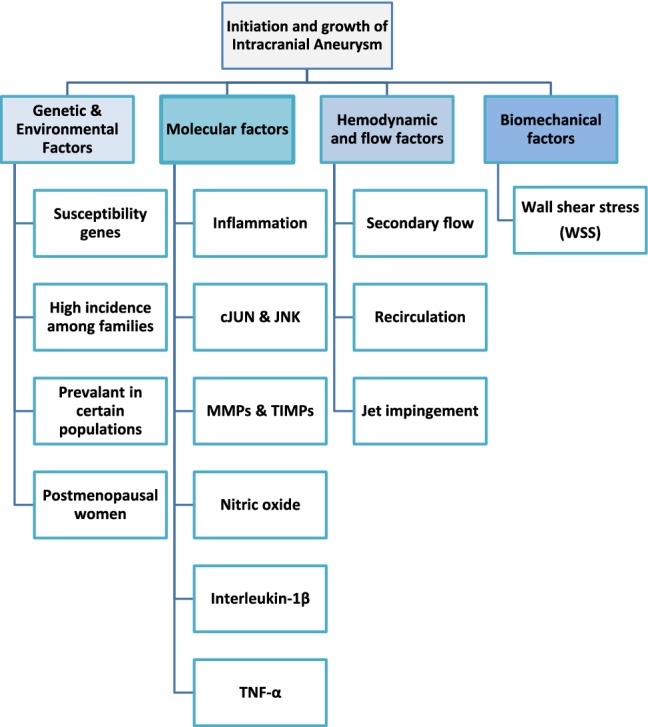
Major parameters involved in the initiation and growth of an IA (Francis et al. 2013)

IAs may occur in the four main brain arteries of the internal carotid artery, the anterior cerebral artery, the middle cerebral artery, and the vertebrobasilar or posterior artery. The general view of IAs generation is that in addition to the genetic, environmental, molecular, and hemodynamic flow factors, shear stress is the only biomechanical factor for initiation and enlargement of brain aneurysms (Francis et al. 2013). Arterial wall remodeling, as a complex interaction between biomechanical and biochemical factors (see Fig. 1), is the key phase of initiation, progression, and also rupturing of aneurysms. But inflammation is the first step in the formation of an IA that starts with the penetration of inflammatory cells (monocytes, macrophages, neutrophils, and lymphocytes) into the vessel wall structure. These micro-phase changes are affected by hemodynamic stress changes in the vascular wall that lead to the effect of the intimal endothelium and remodeling of the local vessel wall. The result of this phenomenon is that it initiates aneurysm formation and the endothelium dysfunction causes the aneurysm enlargement because of the influx of inflammatory cells (Hosaka and Hoh 2014). As a consequence, hemodynamically triggered endothelial inflammatory dysfunction is the main cause of aneurysm initiation.

The parameters that support the role of hemodynamics in the initiation, growth, and rupture of cerebral aneurysms are studied by researchers comprehensively. The main achievement is the fact that the dynamic tensile force resulting from the blood flowing along the surface of the vessel wall, which is described as WSS, is a primary parameter in cerebral aneurysm hemodynamics. Additionally, changes in the magnitude of the WSS vector described by the WSS gradient and the transverse WSS, which is a temporal mean of WSS considering the flow direction over a cardiac cycle, are the other parameters based on shear stress responses in the arterial wall. The vital role of local WSS elevated by turbulent flow patterns in cerebral aneurysm generation has be demonstrated by Sun et al. 2020. They also reported that increasing WSS at the neck and body of aneurysm can be indicated as a parameter for growth of small aneurysms. Furthermore, the effects of the pulse flow, different flow pattern conditions, and tangential force under pulsatile flow are described by the oscillatory shear index (OSI), oscillation velocity index (OVI), and gradient oscillatory number, respectively (Sheikh et al. 2020). Generally speaking, all of these parameters and factors have their own complexity in influence, but most of them are affected by blood flow. Any changes in the blood flow will impact the mechanical responses of the arteries. This is why hypertension is one of the main stimuli for aneurysm formation. The other fact is that hemodynamic factors induce injuries, initiation of inflammation, and remodeling in the arterial wall. There are two major theoretical and conceptual frameworks for the analysis of cardiac circulation effects on endothelial cells: (i) arterial wall enlargement due to transmural pressure (pressure difference between the inside and the outside of a hollow structure) and WSS caused by cyclic strain, and (ii) the frictional reactions of the blood flow that affect the coplanar of the cross section of the artery (Signorelli et al. 2018).

Sometimes, as an undesirable event, blocking with atherosclerotic stenosis can change the physiological scenarios involving blood flow directly, where the most dangerous problem appears as the flow resistance in arteries, which is the major cause of some cardiovascular diseases. In other words, by narrowing the artery, blood flow conditions will tend to change as a result of local turbulence or sluggish perfusion. In this case, not only the biomechanical response of arterial WSS but also hemodynamic characteristics and blood flow forces such as secondary flow, recirculation, and jet impingement may appear that can affect endothelial cells in the genesis and progression of an IA (Jeong and Rhee 2012).

## Method and materials

**Fig. 2:**
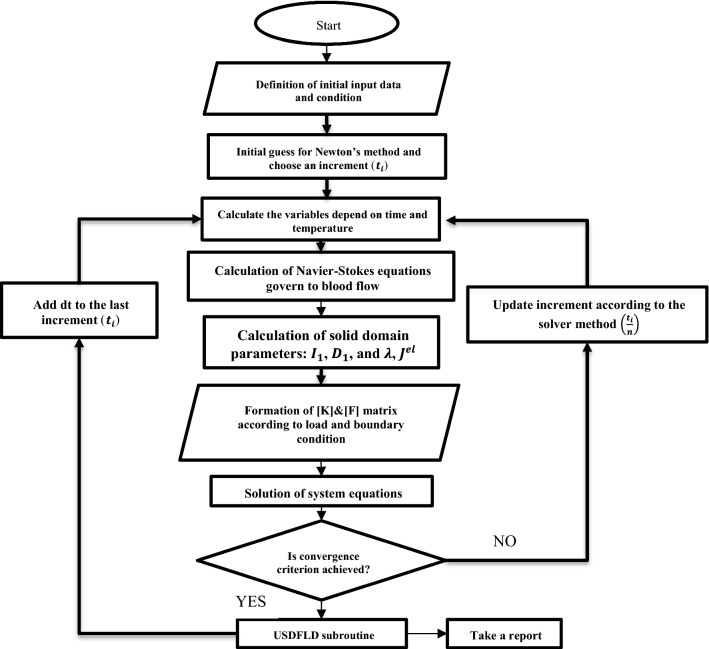
FSI analysis procedure performing by ABAQUS

**Fig. 3:**
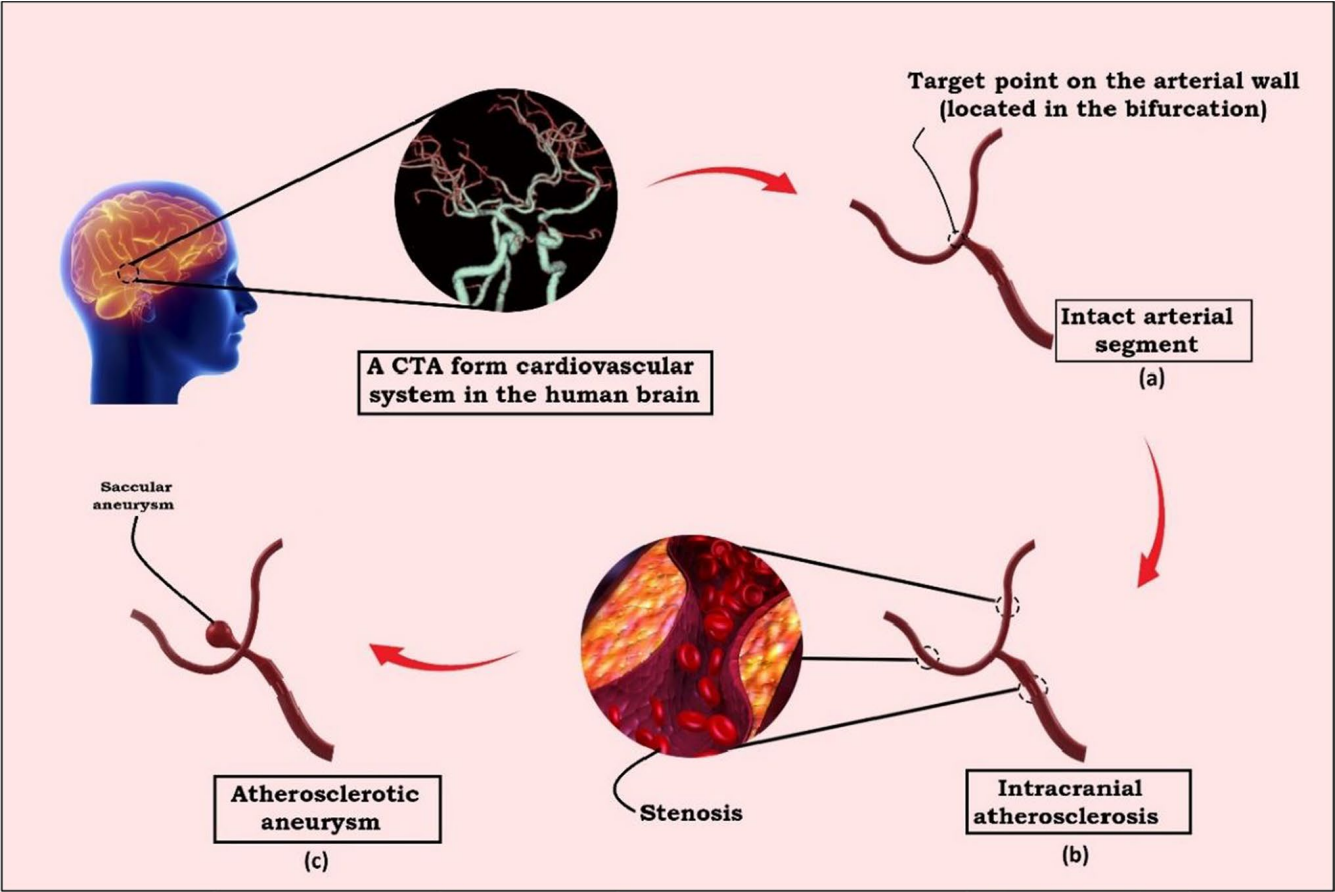
Schematic view of the study conception: **a** 3D geometry of the intracranial artery bifurcation is selected as an intact arterial segment according to a CTA, **b** as the second case of study, the intact arteries are occluded by three atherosclerotic stenoses, **c** the 3D model of an atherosclerotic intracranial aneurysm with a saccular inflammation

**Fig. 4:**
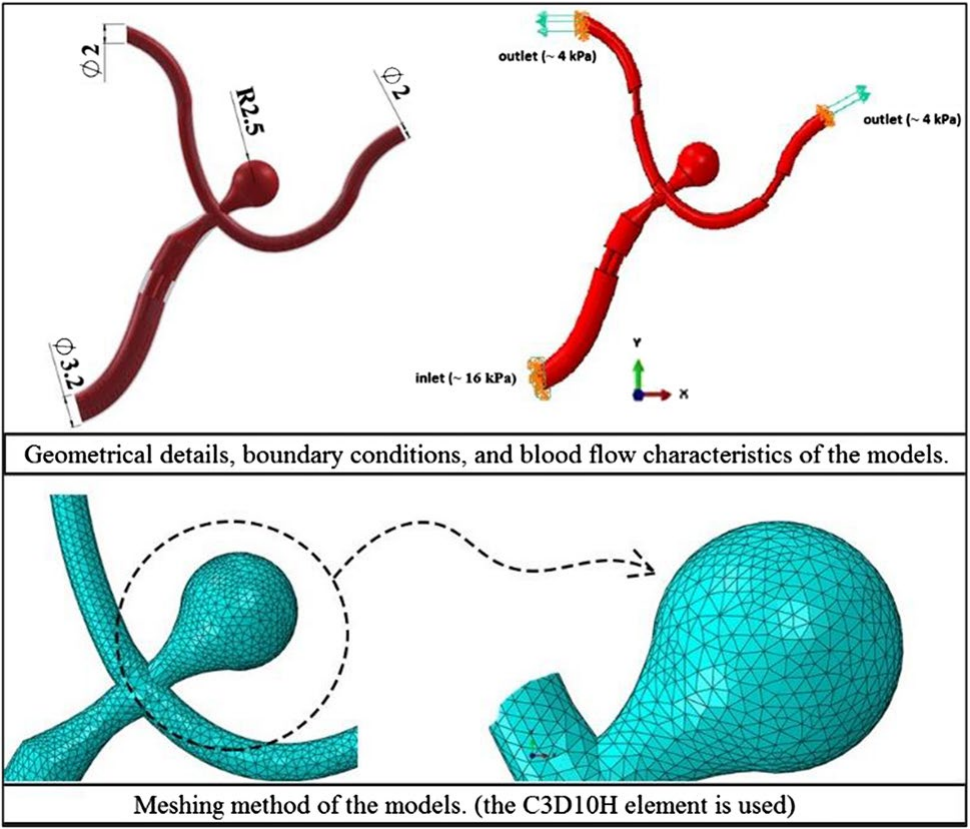
Morphometric, hemodynamic, and FSI simulation details of the studying models

The main purpose of this study is to focus on development and use of advanced FSI computational theories to show how existing atherosclerotic stenosis affects the hemodynamics, initiation risk, and growth of the IAs. However, the theory of FSI is used to consider the interaction between blood and arterial wall tissue domains simultaneously. The majority of previous studies used pure CFD for the study of WSS and OSI distributions in a cerebral aneurysm (Sforza et al. 2009). The main deficiency of the CFD method is a disability in considering the interaction between blood flow and the vessel wall. The resulted stress will come from the fluid pressure, and the displacement structure will be caused by changes in fluid velocity and pressure. Furthermore, as the type of material is hyperplastic, so its displacement is not negligible. While, in the CFD method, it is assumed that the structure is rigid, what’s more, the stress and displacement would not be able to investigate. In fact, FSI study involves CFD and structure analyses simultaneous and it consider their interaction in each increment. In this method, the arterial wall is assumed to be a rigid member, while in reality, these two cases are always in a complex interaction. For this reason, the finite element program package of ABAQUS is used to create the corresponding FSI models. Figure 2 illustrates the following FSI analysis procedure for this project. Also, all case descriptions, geometrical properties, and simulation procedures have followed the studying conception steps mentioned in Fig. 3. The dynamic implicit solver and transition flow are chosen for the simulation of the artery blood flow, respectively. A total time step of 0.1 s is considered. One of the main issues in this modeling procedure is defining the fluid–structure co-simulation boundary for flow and artery. For this purpose, the same coordination should be used for both of them. Considering the FSI theory for the models is performed by a key FSI modeling assumption: The vessel deforms under the action of blood flow, and then, the blood pressure changes are applied to the vessel wall. As it is shown in Fig. 4, the geometry of all three models is based on a general concept with a parent artery (simulated with a 3.2 mm diameter) that contains bifurcation support with two vessels of 2 mm diameter. By the fact that the influence of intracranial atherosclerosis in small cerebral vessels is not completely proven (Boulouis et al. 2016), the mentioned sizes are selected for the radius of the arteries. But for the ACA model, the only difference is a saccular aneurysm with a 2.5 mm radius of inflammation that is located at the bifurcation point. Also, 125 µm and 27 µm are applied to the models for arterial and aneurysm wall thickness, respectively.

### Blood flow and hemodynamics of the models

The Reynolds number of blood flow in the arteries is low, and it is considered to be laminar. Also, the Navier–Stokes equations govern blood flow as an incompressible flow. The Arbitrary Lagrangian–Eulerian formulation is used, which is a common approach for vascular blood flow applications (Nobile 2001; Gerbeau et al. 2005). Rheology studies prove that the blood is a non-Newtonian fluid (Thurston 1979). So, the non-Newtonian properties of blood are considered in this research, and the Herschel–bulky model is employed for this purpose. Equation 1 illustrates the Herschel–bulky approach in which η, γ ˙, κ, *n* and τ_0_ denote the effective viscosity, shear strain rate, consistency factor, power-law index, and yield shear stress, respectively (Campo-Deaño et al. 2015).

1 $$\eta = \kappa \dot{\gamma }^{n - 1} + \frac{{\tau_{0} }}{{\dot{\gamma }}}$$

where $\kappa = 8.9721 \times 10^{ - 3} \frac{{{\text{Ns}}^{{\text{n}}} }}{{{\text{m}}^{2} }}$, $n = 0.8601, \tau_{0} = 0.0175\frac{{\text{N}}}{{{\text{m}}^{2} }}$, density = 1020 $\frac{{{\text{Kg}}}}{{{\text{m}}^{3} }}$, viscosity = 0.04 $\frac{{\text{g}}}{{{\text{cm.s}}}}$,

### Boundary conditions

Inlet and outlet boundary conditions are defined by the velocity and pressure profiles, respectively. These important factors have been derived from the experimental work by (Abdi et al. 2016) and (Liu et al. 2015). No-slip boundary conditions have been set for the fluid–solid interface. Traction compatibility conditions are selected at the fluid–solid interface. Also, the surface of inlet and outlet is constrained in all component’s directions. Accordingly, one inlet velocity of 0.3 m/s with a 16 kPa (~ 120 mmHg) inlet pressure and two outlets pressure of 4 kPa are introduced for the creation of the boundary conditions of the models (see Fig. 4). The boundary condition on the flow should be also determined. In this study, there is a parabolic profile distribution for the flow and velocity and it is assumed that the flow is steady and fully developed with the mean flow of 0.23 m/s. Furthermore, the Reynolds number is 223 that shows a laminar flow (Ortega et al. 2007).

### Meshing method

For the models, there are several alternatives to choosing a meshing type. But the (C3D10H) element (that is a quadratic tetrahedron, hybrid, and constant pressure element where the calculation is considered in 10 nodes of element) is selected for the arterial tissue meshing. The C3D10 and C3D10HS are both 10 node tetrahedral elements in Abaqus with quadratic interpolation of displacement, otherwise colloquially referred to as “2nd order Tets”. While the C3D10 is the standard or “vanilla” 2nd order Tet element, C3D10HS is marketed as a 2nd order Tet element with improved surface stress visualization (see Fig. 4). Also, the CFD element of (FC3D4) with a linear fluid tetrahedron element including 4 nodes in each corner is considered for blood fluid meshes. The number of elements and nodes are reported in Table 1 for three case studies: an intact arterial segment, an artery occluded by atherosclerotic stenosis, and an ACA segment. In addition, the mesh sensitivity analysis is performed for the first case to check the meshing and boundary conditions validity.

**Table 1:** The node and element number of utilized mesh

Models	Fluid/Solid Domain	Number of elements	Number of nodes
Intact intracranial artery	Fluid	33,975	7620
	Solid	25,106	8205
Artery with ICAS	Fluid	33,154	7557
	Solid	27,756	8700
Atherosclerotic aneurysm	Fluid	42,780	9563
	Solid	32,266	10,207

### Arterial tissue modeling

Arterial tissues, because of their structural component supported by collagen, are known as soft tissue with hyper-elastic material behavior. The biomechanical behavior of soft tissues is complex and often difficult to characterize. But the elastic properties of the artery and aneurysm wall were determined by Scott et al. (Scott et al. 1972). The blood and arteries are always in interaction and the pressure of blood causes radius deformational changes in arteries. The blood pressure and artery radius change have a nonlinear relationship (Zulliger et al. 2004). Soft tissue is composed of fibers and matrix. In this study, the fibers and matrix are assumed to be incompressible materials. Hence, the tissue is considered an isotropic hyper-elastic material.

The neo-Hookean hyperplastic model is considered for defining the behavior of the artery. A recent study performed by (Parshin et al. 2019) proves that this method is the optimal approach for modeling the mechanics of cerebral aneurysm wall and solving the FSI problem. For large strains, the Neo-Hookean model provides an accurate description of deformation and is shown to be suitable where strains are up to 20%. Although the hyperplastic material is orthotropic, for the sake of simplicity, an isotropic behavior is assumed in this work that will result in an acceptable accuracy. The strain energy density function for this model can be calculated by Eq. 2:

2 $$W = C_{1} \left( {I_{1} - 1} \right) + \frac{1}{{D_{1} }}\left( {J^{el} - 1} \right)^{2}$$

and the uniaxial stress is:

3 $$\sigma = 2C_{1} \left( {\lambda - \frac{1}{{\lambda^{2} }}} \right) + \frac{2}{{D_{1} }}\left( {J^{el} - 1} \right)$$

where $C_{1}$, $I_{1}$, $D_{1}$, $\lambda$, and $J^{el}$ are linear part of elastic energy, invariants, temperature-dependent material parameter, elastic volume ratio and stretch ratio respectively. $C_{1}$ and $D_{1}$ are assumed as follows:

4 $$\begin{gathered} \left\{ {\begin{array}{*{20}l} {{\text{C}}_{1} \left( {{\text{linear}}\,{\text{part}}\,{\text{of}}\,{\text{elastic}}\,{\text{energy}}} \right)} \hfill & \to \hfill & {166\,({\text{kPa}})} \hfill \\ {D_{1} } \hfill & \to \hfill & 0 \hfill \\ \end{array} } \right. \hfill \\ \tau = 2\mu .\left( {\dot{\varepsilon }.\vec{n}} \right) \hfill \\ \end{gathered}$$

where $\dot{\varepsilon }$ is the rate of deformation tensor, $\vec{n}$ describes the inward normal vector, and μ is the blood viscosity.

### The formulation for wall shear stress calculation

In non-pulsatile flow in a straight vessel, fluid does not move at the same velocity at every point in the vessel. Instead, fluid flow is fastest at the center and slowest close to the wall. The fluid velocities assume a parabolic profile referred to as the "laminar flow" profile. This pattern of flow is the result of friction within the fluid and between the fluid and the vessel wall, and is related to the fluid viscosity. This friction creates a tangential force exerted by the flowing fluid and is referred to as the "wall shear stress". The magnitude of wall shear stress depends on how fast the fluid velocity increases when moving from the vessel wall toward the center of the vessel. This velocity gradient near the wall is called the wall shear rate. For calculating the WSS vector in every point of the arterial wall, formulating the FSI analysis with Eq. 4 is required. Also, Eq. 4 can be expanded for preparing the needed subroutine as Eq. 5:

5 $$\tau = 2\mu \left[ {\begin{array}{*{20}c} {\frac{{\partial v_{x} }}{\partial x}} & {\frac{1}{2}\left( {\frac{{\partial v_{y} }}{\partial x} + \frac{{\partial v_{x} }}{\partial y}} \right)} & {\frac{1}{2}\left( {\frac{{\partial v_{z} }}{\partial x} + \frac{{\partial v_{x} }}{\partial z}} \right)} \\ {\frac{1}{2}\left( {\frac{{\partial v_{x} }}{\partial y} + \frac{{\partial v_{y} }}{\partial x}} \right)} & {\frac{{\partial v_{y} }}{\partial y}} & {\frac{1}{2}\left( {\frac{{\partial v_{z} }}{\partial y} + \frac{{\partial v_{y} }}{\partial z}} \right)} \\ {\frac{1}{2}\left( {\frac{{\partial v_{x} }}{\partial z} + \frac{{\partial v_{z} }}{\partial x}} \right)} & {\frac{1}{2}\left( {\frac{{\partial v_{y} }}{\partial z} + \frac{{\partial v_{z} }}{\partial y}} \right)} & {\frac{{\partial v_{z} }}{\partial z}} \\ \end{array} } \right].\vec{n}$$

where $v$ shows the blood velocity in the calculation of wall shear stress. Indeed, variation of this term with displacement expresses shear stress.

The USDFLD subroutine is set for observation of the WSS changes throughout the arteries. The written subroutine enables the definition of variables at a material point as functions of time for any of the available material point quantities. In fact, the variation of velocity will be called for all material points of elements for which the material definition includes user-defined field variables and then put in the WSS equation for calculating this parameter.

## Results and discussion

In this study, three different scenarios of the human cerebral artery segment are simulated, considering the interactions between blood flow and the arterial wall tissue. As it can be seen in Fig. 3a, in the first step, a healthy cerebral artery is modeled to observe the hemodynamics of the bifurcation during a cardiac cycle in physiological condition. The main purpose of modeling a segment of a healthy cerebral artery is to see in which patterns the biomechanical changes are observable during a normal cardiac cycle. Then, it will be possible to use the WSS, velocity, displacement, and other hemodynamic parameters as indicators for performing comparative analysis between an intact artery and two other remodeled vessels. In the next steps, a cerebral artery containing plaque occlusions (with different ratios of the arterial section to stenosis area) is simulated in completely similar analysis conditions firstly without aneurysm (see Fig. 3b) and then includes a saccular aneurysm (see Fig. 3c). Finally, all numerical and graphical outputs are collected for three corresponding models separately. Despite the fact that the bifurcations are vulnerable parts of the arterial system (Fukuda and Aoki 2015), it has been demonstrated that due to probable changes in blood-flowing patterns, dynamic responses on the arterial wall located at this point may appear as determinative factors for tissue remodeling. However, all dynamic responses for giving statistical results are preferred to be taken from this critical area. As it is shown in Fig. 3a, the study target point elements are determined at the middle of the arterial wall located at the bifurcation and the vertex point of the sac for the models without an aneurysm and for the ACA model, respectively.

### Biomechanical and hemodynamic effects of atherosclerosis

**Fig. 5:**
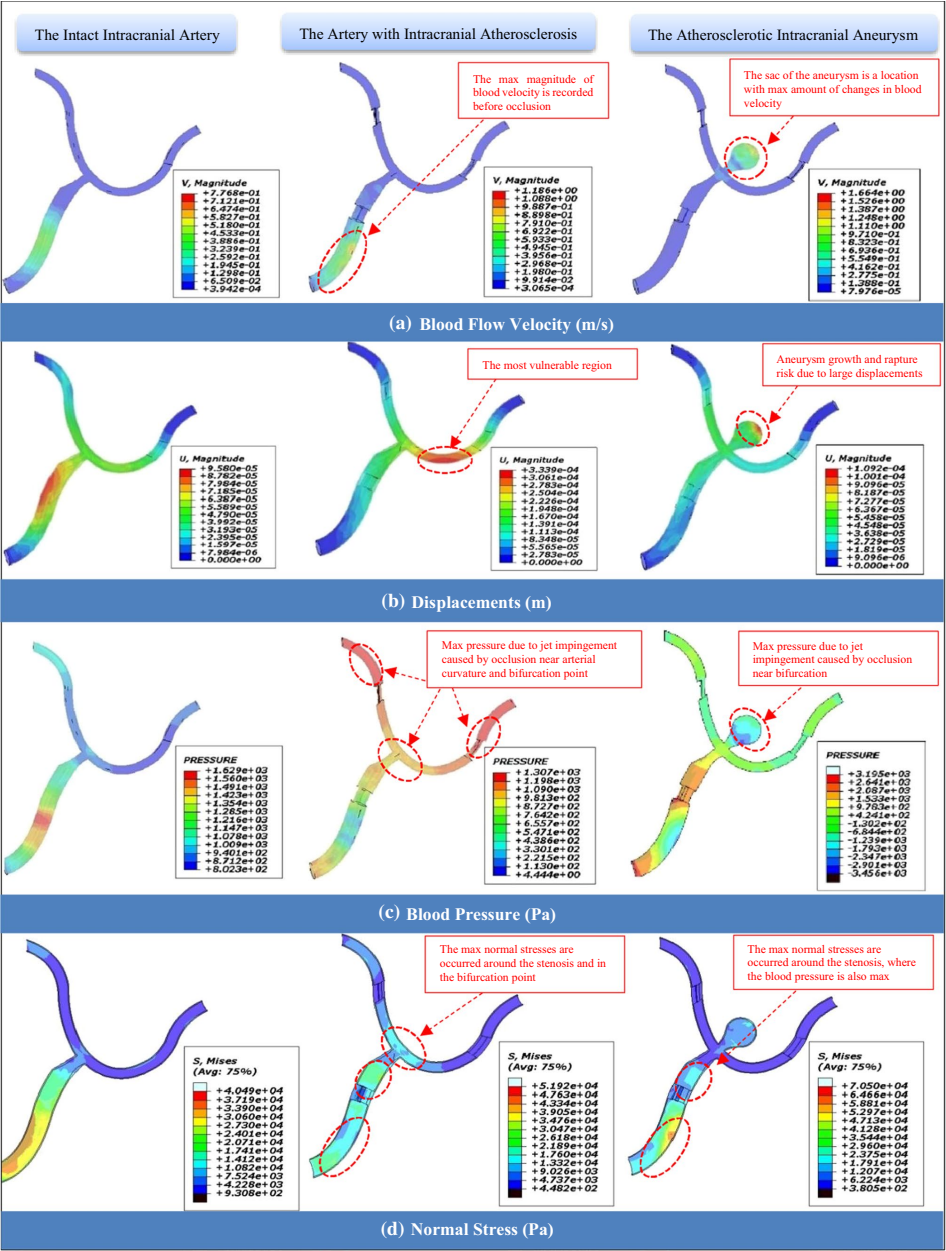
Graphical results for biomechanical responses and hemodynamics of the models: **a** the velocity changes throughout the arteries, **b** arterial tissue displacements due to hemodynamic changes during analysis, **c** effective blood pressure on the arterial tissue from the cardiac cycle during analysis, and d) the normal stresses measured throughout the arterial walls

**Fig. 6:**
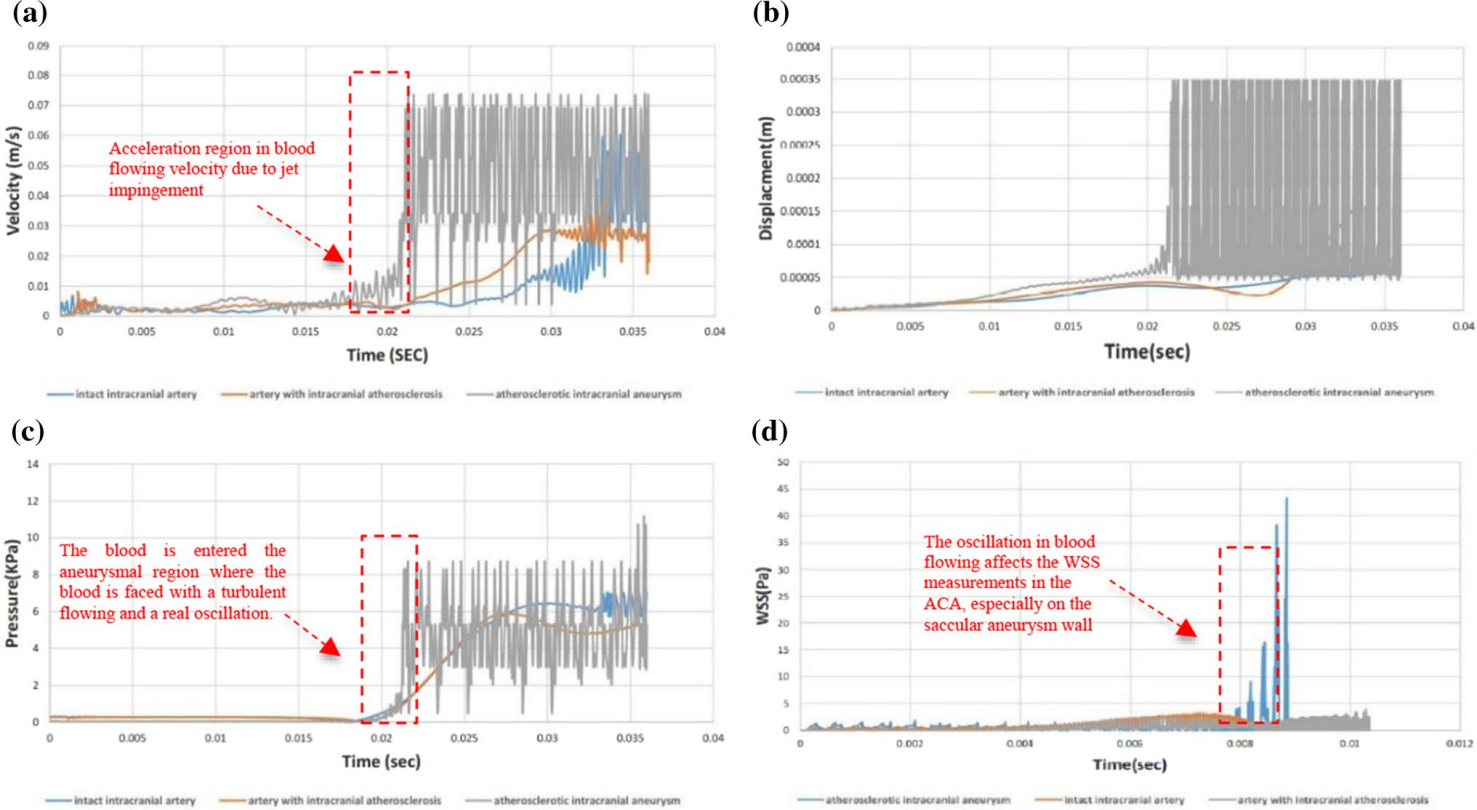
Statistical data of numerical results recorded in the target point for biomechanical and hemodynamic responses: **a** blood velocity, **b** displacements and deformation, **c** blood pressure measured in target point (the bifurcation point), **d** WSS variations recorded for target points (the vertex point of the sac)

**Fig. 7:**
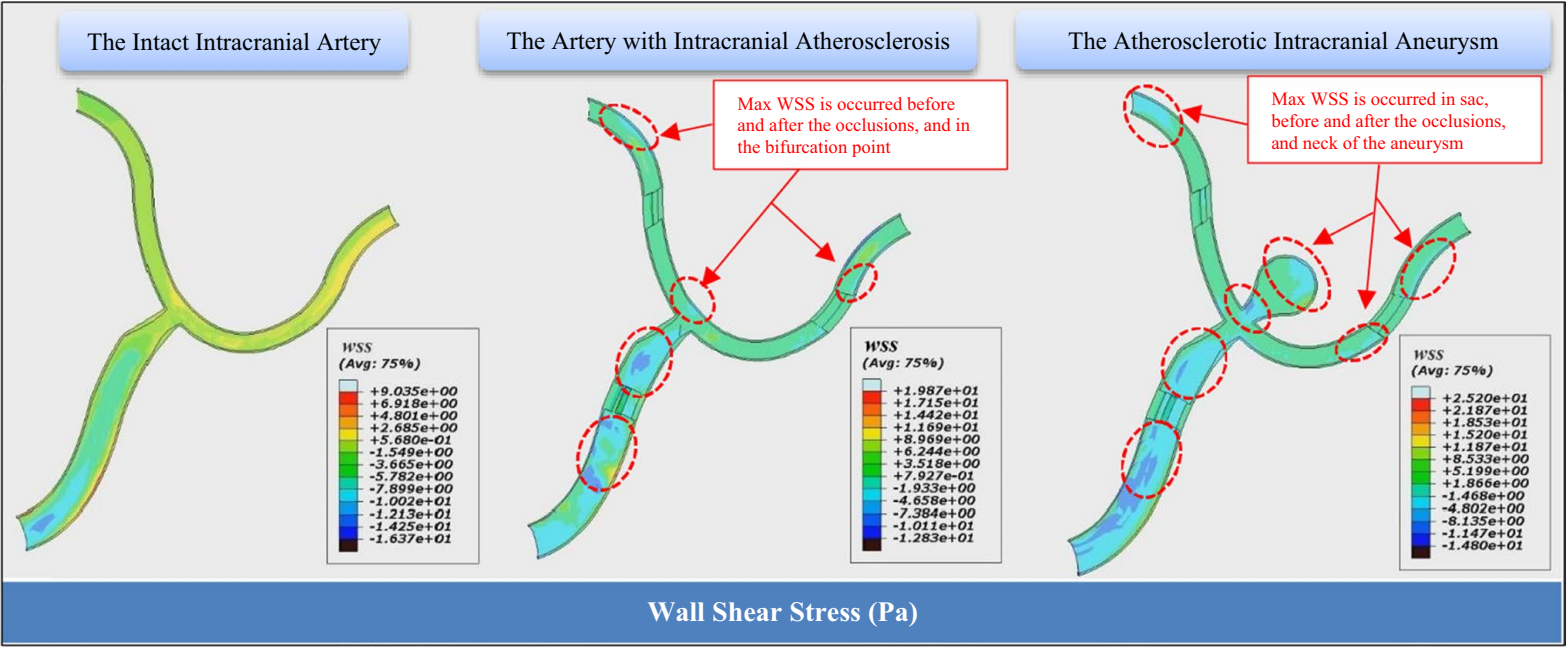
Wall shear stress obtained magnitudes for three assumed scenarios

The significant advantage of using an FSI analysis in vascular engineering is that it takes into account the impact and interaction of the blood flow on arterial tissue and vice versa. Because of the way the FSI analysis was performed in this study, both biomechanical and hemodynamic responses in the arterial wall of the models are available. The translational responses of the blood velocity in the modeled arterial segments (Fig. 5a), arterial tissue displacements due to mechanical impacts of blood flow during analysis (Fig. 5b), the measured blood pressure throughout the vessels that is illustrated in Fig. 5c, and the normal stresses recorded on the arterial walls (Fig. 5d) are recorded for target points in a time range of (0 to 0.036 s). The mentioning parameters are observed for the whole of the models but for the target points, despite preparing the graphical results the statistical outputs are also presented in Fig. 6. Besides, the most important factor, WSS, is also recorded for all models and reported separately as shown in Fig. 7. The intact model experiences laminar flow, which means that the flow is periodic but stable (Yagi et al. 2013), in a normal physiological condition till the inlet pressure of the artery is in a constant and healthy range. However, the significant increase in deformation magnitudes can be seen when the blood flow is turbulent due to stenosis. Any morphological changes in the cross-sectional area of the vessel lead to the formation of a jet impingement flow, whereas most likely for the intact artery model, after about 0.02 s of the analysis, the deformation of the arterial wall tends to grow slightly after showing the median displacement magnitude of 0.05 mm. By experiencing a focal impinging jet (Cebral et al. 2005), the blood flow velocity is observed at a maximum amount located in the artery before occlusion in the parent artery. These observations are shown in Fig. 5a and b for the blood velocity changes and displacements in the models, respectively. Despite having similar patterns, magnitudes in the time history of the performed FSI analysis are completely dependent on fluid velocity and tissue deformation. When the artery is under dynamic loading from a turbulent blood cycle due to cross-sectional area blocking, the mechanical response of displacement in the arterial wall located after occlusion may rise significantly. It is obvious from Fig. 5b that the maximum magnitude of displacement in the arterial wall tissue can occur in a region outside of the bifurcation point. In other words, despite considering the bifurcation point as a vulnerable region and target point in the hemodynamic analysis, it is observed that the curve region of the vessel is records a high amount of deformation. In the region after occlusion, in the distance between the head of the stenosis and the bifurcation point, the velocity tends to increase significantly where the jet impingement occurs. Although the vessel wall exhibits stretch with a significant amount of 0.20 mm at the bifurcation point, conditions may be prepared for initiation of arterial remodeling. This important result could be demonstrated by observations of maximum blood pressures, as shown in Fig. 5c, in both arterial curved and bifurcation regions located after the stenosis due to jet impingement. Not only is the WSS responsible for the genesis of an aneurysm but the stretch is also another determinative biomechanical parameter. Considering the target point on the aneurysm sac wall, the maximum magnitude of deformation is recorded at about 0.11 mm for the ACA model. Notwithstanding a low magnitude in comparison with the model without an aneurysm, the vulnerable magnitude of deformation is in a low range for aneurysm wall because of the low thickness in this region. Although both velocity and deformation have an upward trend from the neck of the aneurysm to the vertex point of the sac, there is no exact indicator for estimation of the rupture risk in the aneurysms, but any large stretch in the saccular wall may lead to rupture in the aneurysm. Also, for the artery with ICAS, the maximum blood pressure is observable due to jet impingement caused by occlusion near arterial curvatures and bifurcation points (see Fig. 5c). Passing throughout the blocked regions, the blood velocity is increased following this phenomenon, and the significant changes in jet impingement flow lead to maximum pressure being applied to the arterial walls. In the hemodynamic view, blood flow is the main cause of both pressure and WSS stress. The pressure acts on the lumen as normal stress, which affects the endothelium, physiology, and the smooth muscle cells (as tension). Following the blood pressure variations throughout the arteries, the normal stress changes on the arterial wall are in similar patterns. It is obvious that maximum amounts of the normal stress are observed around the stenoses, where the aggregation of the blood leads to a turbulent flow. Again, the bifurcation point is faced with considerable normal stress on the wall. This periodic oscillation may apply impact loads during acceleration in blood velocity and blood pressure (see Fig. 6a and c, respectively).

Generally, the effects of atherosclerosis on the behavior of the vascular tissue can be observable from both biomechanical and hemodynamic aspects. Existing occlusion in the cross-sectional area of the vessel changes the blood flow patterns throughout the involved vascular segments. Following this, arterial tissue endures abnormal oscillations of tissue deformation and stretch, blood pressure and velocity, and stresses. All of the mentioned phenomena affect the micro-mechanical phase of the endothelial cells, leading to arterial tissue remodeling and inflammation.

### The impact of atherosclerosis on WSS, the arterial tissue remodeling, and aneurysm initiation

The primary signals for inflammation in arterial wall tissue are shear and normal blood flow stresses. Shear stresses are the main cause of degeneration of the elastic lamina and endothelial damage in arteries that are the first stage of tissue remodeling. These mechanical responses affect the intimal endothelium in micro-phase changes that lead to local arterial wall remodeling.

In normal cardiac circulation, shear forces that depend on blood flow rate and blood velocity appear as frictional forces on the inner vessel wall. WSS is related to applying force per unit area by blood flow and/or the result of viscosity, layer friction in a turbulent flow with different velocities, and shear rate in a tangential direction to the arterial wall. The aforementioned changes occur near bifurcations which are zones with a great potential of structural function and modulation in endothelium by hemodynamic forces (Hosaka and Hoh 2014). Experimental studies have demonstrated that the high WSS combined with a high positive WSS gradient can increase arterial remodeling at the arterial bifurcation (Meng et al. 2007). Taylor and Humphrey 2009 demonstrated that the arterial wall can be assumed to be a hyper-elastic material in most physiological cases due to its exhibiting viscoelastic behavior because of its having elastin and fibrillar collagen, a ground substance matrix, and resident cells in its complex composite structure. Furthermore, Malek et al. (Malek et al. 1999) presented a range of WSS magnitudes (for low-shear and high-shear index) encountered in arteries in a physiological condition for normal arteries between (1 and 7 Pa). As it can be seen in Fig. 7, the observable maximum range of the WSS in the intact intracranial artery is based on the WSS range for an artery in physiological conditions. The most considerable result is obtained for the WSS variations recorded in the target points for both intact and ICAS artery models (see Fig. 6d). In fact, the WSS magnitude for the bifurcation point is recorded at less than 5 Pa.

On the other hand, the initiation of an IA is a complex interaction among biology, biomechanics, and hemodynamics, but the association of abnormal hemodynamics is determinative.

The local protease activity (an enzyme that breaks down proteins and peptides) and the collagen synthesis rates, which are controlled by cellular responses to hemodynamic loads, are two important parameters that affect arterial wall strength (Taylor and Humphrey 2009). The loss of mechanical strength and stretch of the arterial tissue due to changes in blood pressure are the basis for starting the arterial tissue remodeling procedure. In addition, for the explanation of the pathogenesis of IAs, the variation in WSS in the lumen is another vital factor besides the luminal forces. But for the role of atherosclerosis in the initiation of IA, the alteration in WSS due to jet flow can be controversial. Although Kono et al. 2013 demonstrated that the degrees and location of stenosis affect the WSS directly, it may change the IA initiation scenario with developing unphysiologically high WSS, wherein WSS can be measured as more than double of the physiologically indexed amount near the impingement zones.

By the fact that there is no exact definition for the physiologic range of WSS in IAs (Cebral et al. 2017), the determination of a comparative range for computational studies is also a real challenge. However, the WSS results for three models reported in Fig. 7 present interesting observations. The ICAS model shows the peak of the WSS in the arterial wall located after stenoses, where the jet flow has occurred, and the target point on the bifurcation. In this model, the WSS amount increases slightly from the intact arterial region (with WSS < 7 Pa) to the first stenosis in the parent artery. But the peak of the WSS (19 Pa) is recorded around the occlusion regions. Obviously, the bifurcation point and the arterial curvatures located after the stenoses are the most vulnerable points for IAs initiation. This probability may be supported by the measured roughly doubled amount of the WSS in the mentioned regions. A similar scenario is observable for the intracranial ACA model, but the only difference is that the neck and vertex of the saccular aneurysm are affected by a large amount of the WSS (25 Pa). The obtained WSS measure is a significant peak for a saccular IA which may be a cause of aneurysm rupture. Furthermore, the high WSS around the stenoses predisposes the arterial wall to genesis an inflammation for initiation of fusiform aneurysms. Overall, the role of two important factors, stenosis location and degree in atherosclerosis effects on IA initiation is undeniable. For example, because of fluid dynamics, the existence of a stenosis at a more distance from the bifurcation will apply less blood velocity due to jet flow in comparison with a model in which the stenosis is close to the bifurcation.

## Summary and discussion

Narrowing of the cerebral vessels will lead to unexpected changes in the blood flow patterns. These changes may occur in blood velocity and pressure, which are important parameters in biomechanical stability of the vascular system. The FSI analysis shows that consideration of the interaction between blood flow and arterial tissue is a vital factor for studying arterial tissue remodeling caused by atherosclerosis. As the investigation of the impact of atherosclerosis on the hemodynamics and mechanics of the vessels has been demonstrated in previous sections, the significant changes are observable in arterial bifurcations and curvatures (Bonneville et al. 2006). In fact, the results show that arterial bifurcations and the outer walls of arterial curvatures are the most vulnerable locations for the initiation of an IA. This is the major reason for designing the morphometric assumptions of the corresponding models. The variation in normal stress applied by the blood pressure and frictional impacts of the blood flow as shear stress on the arterial wall play a significant role in endothelial cell destruction. On the other hand, researchers believe that shear stress is a determinative factor in inflammation of the vessel wall. The peak of the WSS can be seen when the distance of the occlusion is low from the bifurcation wall, where the jet flow can affect the wall tissue considerably. Hence, the important observation is demonstrated that the impact of the atherosclerotic factors on IA initiation is location dependent. Furthermore, stenoses close to the arterial curvatures are the main risk factors for tissue remodeling initiation. Despite the impacts of vessel narrowing on aneurysm genesis, likewise, it may be considered a risk factor for aneurysm rupture. This is demonstrated that increasing the blood flow velocity into the IA applies unexpected shear and normal stresses to the saccular wall. Although the conception of the coiling treatment method for IAs is embolization of the saccular section to eliminate blood flow turbulence, oscillation in the blood velocity due to atherosclerosis may be one of the risky factors in surgical complications.

## Conclusion

In this study, FSI analyses have been performed to show how existing stenoses could affect and predispose an intact intracranial artery to aneurysm initiation considering the blood and arterial tissue interaction. After the simulation of three different scenarios of a cerebral artery segment, the multi-physical FSI analysis results were studied in both statistical and biomechanical aspects. According to the recorded results, the significant influence of atherosclerosis in arterial tissue remodeling of cerebral arteries is demonstrated. Also, the turbulent blood flow caused by stenosis plays important role in demonstrating the direct effect of atherosclerosis in the hemodynamic changes leading to arterial tissue remodeling and IA initiation. Although there is no exact definition for biomechanical variation impact ranges in aneurysm rupture, the authors' future studies will be focused on the effects of atherosclerosis on the IA rupture.
